# The beginnings of internal fixation of fractures in the pre-aseptic period 1797–1870

**DOI:** 10.1007/s00264-026-06895-z

**Published:** 2026-06-12

**Authors:** Jan Bartoníček, Ondřej Naňka

**Affiliations:** 1 Department of Orthopedics, First Faculty of Medicine, Charles University and the Central Military Hospital, Prague, Czech Republic; 2 Institute of Anatomy, First Faculty of Medicine, Charles University, Prague, Czech Republic

**Keywords:** History, Fractures, Internal fixation, Infection

## Abstract

The first successful internal fixation in the pre-aseptic period was performed by the Flemish surgeon Dominique Le Roy in 1796 in Antwerpen, using a gold wire in a patient with an open fracture of the lower leg. In the first half of nineteenth century, he was followed by a number of other surgeons, among them Achille Cléophase Flaubert, father of the famous novelist Gustave Flaubert, who treated both acute fractures and non-unions. Almost all operations resulted in suppuration, however, in many patients the fracture healed, while other cases ended in amputation or even death. Despite a series of failures, individual operations inspired the next generation of surgeons. Nevertheless, it took another 40 years before the foundations of modern internal fixation were laid by the Belgian surgeon Albin Lambotte.

## Introduction

History of surgical treatment of fractures has been the focus of numerous studies [1–21]. These studies have addressed both general internal fixation techniques [2, 5, 7–10, 15–19, 21] and internal fixation of specific fractures [12–14], particularly fractures of the proximal femur [1, 3, 4, 6, 11, 20]. Until the mid-nineteenth century, the obstacles to the development of surgical treatment of fractures included primarily pain, infection, and the inability to visualize the actual fracture, i.e., the non-existence of radiological imaging methods. Morton’s discovery of ether anaesthesia in 1846, the introduction of Lister’s method of antisepsis in the 1870 s, and, above all, the method of asepsis, gradually developed by German surgeons, resolved the first two problems [10]. Röntgen’s discovery of X-rays in 1895 represented a fundamental breakthrough not only in the diagnosis of fractures but also in the assessment of treatment outcomes [5, 10].

However, some daring surgeons did not wait for these discoveries and attempted to stabilize fractures surgically even before the introduction of antisepsis and ether anaesthesia. Although this history was examined by a number of authors [2, 5, 7–10], the facts they present are not always accurate, and some of them are missing. Furthermore, new, previously unknown details have emerged recently, that have changed our view of certain important historical milestones [22].

### The first mentions

The first reported case of internal fixation is sometimes attributed to the humeral fracture treated with wire cerclage in 1775. A detailed analysis of the original sources has shown that the actual events unfolded somewhat differently [22]. It was a discussion between two French surgeons, namely ***Alexis Pujol (1739–1804)*** and ***Jean-Francois Icart (1734–1803)***, in the journal J. Med. Chir. Pharm (Paris 1775). Pujol indirectly accused Icart of causing the patient’s death from sepsis following the cerclage procedure he performed on a humeral fracture. The ensuing exchange of views revealed that this procedure had not been performed at all and that the patient had died from haemothorax. Nevertheless, this was the first time such a treatment option for fractures had been discussed in the professional literature.

### The first internal fixation

A description of what appears to be the first successful case of internal fixation of a fracture was discovered only recently [22]. The Flemish surgeon ***Louis Dominique Le Roy (1755–1826)***, who practiced in Antwerp, successfully treated an open fracture of the lower leg using gold wire cerclage. The patient was a butcher who, on November 28, 1796, fell in the street and suffered an open spiral fracture of the lower leg approximately 12 to 15 cm above the ankle. Initially, he was treated with a bandage, but due to repeated fracture displacements, internal fixation with gold wire was performed. After the operation, antiseptic dressings soaked in various alcoholic solutions were applied. Nevertheless, there occurred suppuration and the sutures gradually loosened. They were removed, and a callus formation was discovered. Approximately 42 days after the operation (February 12, 1797), the skin had healed, and subsequently, function of the limb was restored. Le Roy published his case that same year [23], but in Flemish. This may be why the article was overlooked and was only discovered recently [22].

### The first followers – fractures and non-unions of the humerus—1827–1839

Another detailed description of internal fixation of fractures was published in 1827 by the American surgeon ***John Kearny Rodgers (1793–1851)*** [24]. The case involved a one-year-old supracondylar non-union of the humeral shaft in a 15-year-old boy. Rodgers performed the surgery on July 31, 1827. He excised the non-union, drilled holes in the ends of the fragments, and threaded a wire through the holes (the material is not specified). The wound healed per secundam, and the wire cerclage eventually was forced out of the surgical site. Nevertheless, the fracture healed, although with a 5 cm shortening, and the limb remained functional.

**Fig. 1:**
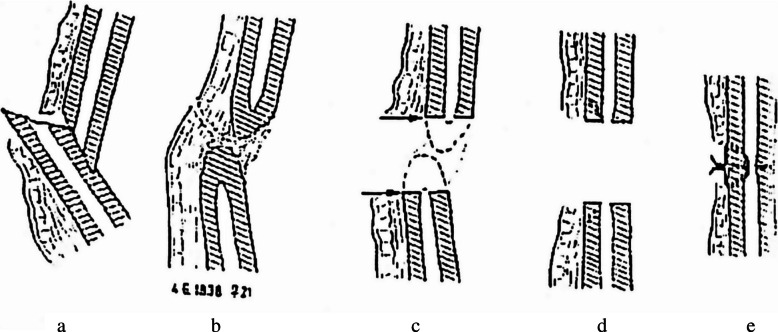
Internal fixation of a humeral non-union performed by Flaubert in 1838 [25]

**Fig. 2:**
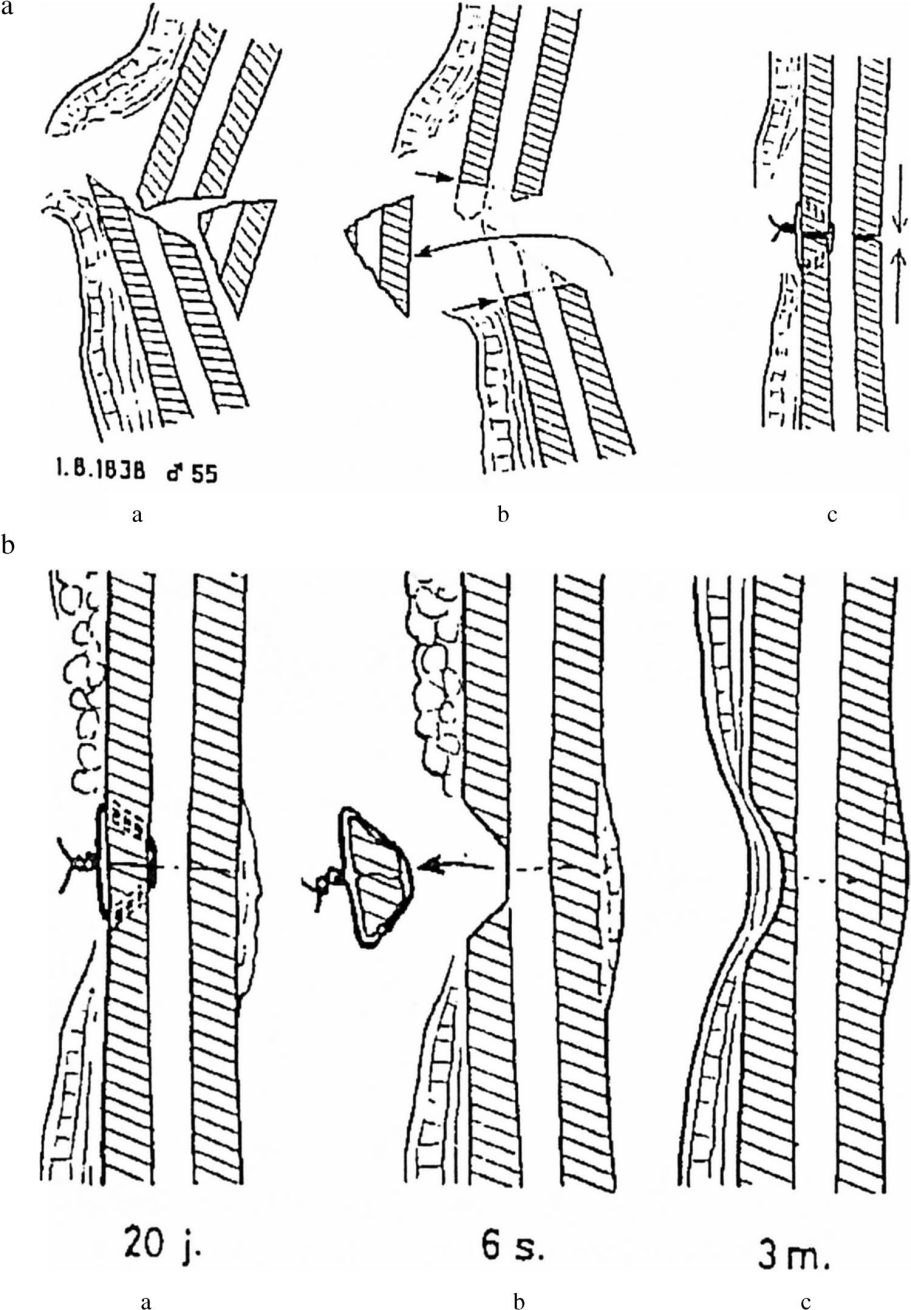
Internal fixation of an open humeral fracture performed by Flaubert in 1838. **a** – technique of osteosuture; **b** – the course of healing [25]

In 1838, ***Achille Cléophas Flaubert (1784–1846)***, the father of the novelist Gustave Flaubert, made his mark on the history of internal fixation [25, 26]. In a 21-year-old woman, he excised a non-union of the humerus and performed internal fixation using unspecified material (Fig. 1). The wound healed per secundam, and temporary paresis of the radial nerve occurred. After the bone sequestrum, including the osteosynthetic material, was removed, the patient recovered. Although a solid bony union was not achieved, the limb remained functional. A few weeks later, Flaubert operated on a 55-year-old coachman who suffered an open fracture of the humerus (Fig. 2). Such injuries were typically indicated for amputation at the time, as they carried a risk of gangrene, sepsis, and death. Flaubert opted for osteosuture performed in a manner similar to the first case. The fracture eventually healed firmly, and the patient returned to his original occupation. Both cases were described by ***Louis-Henri Laloy (1814–1880)*** in 1839 in his *Thesis* [27].

### Fractures of the patella—1841–1868

Patellar fractures were considered a distinct entity due to the bone’s subcutaneous location, and thus its ease of diagnosis. These injuries severely impaired the knee joint function, making internal fixation the obvious choice. However, these were intra-articular fractures, and potential infection—and the sepsis that often resulted from it—sometimes led to the patient’s death. Nevertheless, some surgeons decided to take this risk [14]. According to some sources, ***John Rhea Barton (1794–1871)*** was the first to attempt internal fixation of the patella in 1834 [28, 29]. However, the patient died. The first successful internal fixation of the patella was reportedly performed in 1838 by the American surgeon ***George McClellan (1796–1847)*** [30]. None of these operations, however, were published, and their performance thus remains controversial.

The earliest written reference to surgery for a patellar fracture can be found in an article by the prominent German surgeon ***Johann Friedrich Dieffenbach (1792–1847)***, published in 1841 [31]. The author described a method for treating old patellar fractures involving the transection of the patellar ligament and the tendon of the rectus femoris muscle approximately 7 cm above the patella, followed by the application of a special compression bandage. The aim of the surgery was to allow the fragments to be brought together and to induce an inflammatory reaction that would lead to fracture healing. Dieffenbach reportedly achieved an improvement in the patient’s condition using this method. However, he did not describe any specific case.

In 1843, the French surgeon ***Joseph François Malgaigne (1806–1865)*** published a report on the percutaneous fixation of a fractured patella using a pair of percutaneously inserted hooks, known as “griffes métalliques” [32]. Malgaigne’s method was associated with a number of complications, including infection, subsequent arthritis, and often sepsis with a fatal outcome. ***Henry Mitchell (1826–1910)*** published his experience with Malgaigne’s hooks in two patients in 1861 in the San Francisco Medical Press [33]. The editor’s commentary on the article provides a different description of the treatment: “*Make a longitudinal incision, of sufficient length to expose the fragments; drill the anterior margins of them, with a drill, one line in diameter; then passing a silver ligature through the holes thus made and, crossing the ends and pulling stoutly upon them twisting the end of ligatures together, which holds the fractured portions of the patella in apposition, by which of the a bony union always takes place “* [34]. The author of this article was none other than ***Elias Samuel Cooper (1807–1862)***, a prominent surgeon in San Francisco at the time. Thanks to this publication, Cooper is often cited as the first surgeon to successfully operate on a patellar fracture, even before the introduction of antisepsis. However, in his article, Cooper describes only the method, not a specific case.

The first person to successfully perform internal fixation of the patella before the introduction of antisepsis was most probably ***Thomas Maldrup Logan (1808–1876)***, an American surgeon from Sacramento. In 1864, he performed internal fixation of an inveterate patellar fracture. In February 1864, a 30-year-old man suffered a patellar fracture, which was treated conservatively. Due to the failure of conservative treatment, Logan performed a longitudinal incision under chloroform anesthesia in April 1864, drilled diagonal holes in the fragments through which he passed a silver wire, and connected the two fragments. The wire was removed after six weeks, followed by gradual exercise of movement with very good function. Logan published his case in 1868 [35] and, among other things, mentioned Cooper in his article.

### Non-unions of the femur—1843

**Fig. 3:**
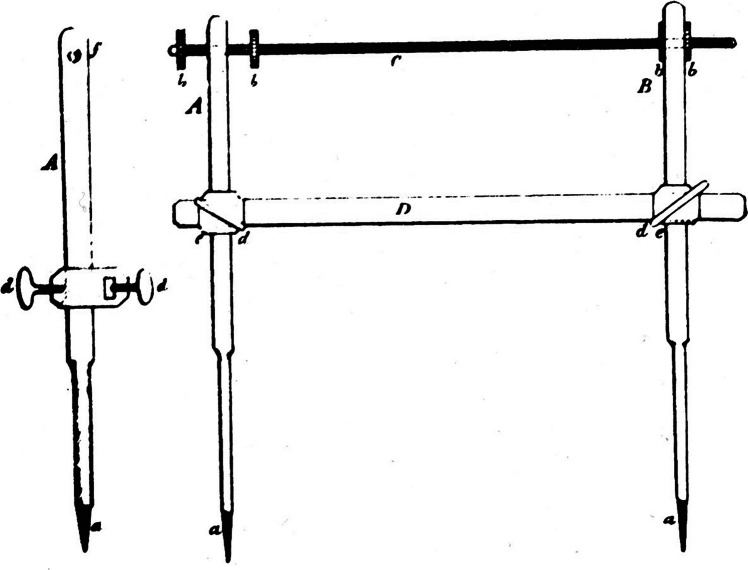
External fixator used at the Wutzer´s department [8]

In his 1847 dissertation thesis, the German physician ***François Charles Joseph Hubert Geller (?-?)*** examined two cases of surgical treatment for non-union of the femoral shaft [36]. The first operation was performed by ***Claus von der Höhe*** in February 1843. The patient was a young man with an unhealed femoral fracture. The aim of the operation was to excise the non-union and fix it using a device called the “Tirefond.” According to the description, it was a simple fixator consisting of two screws, each screwed into one of the main fragments and connected by a transverse rod (Fig. 3). However, the bone quality was poor, and the screws did not hold securely. The postoperative course was complicated by arterial bleeding and sepsis. The patient died a few weeks later. In the second case, also involving a young man, the operation was successful. The procedure was performed on March 24, 1846. The report does not mention the surgeon’s name. The bleeding fragments of the femur were fixed by a gold wire. Despite subsequent suppuration, the non-union healed within seven months after the operation. The first case mentioned is discussed in the historical literature [8] as the first use of external fixation. In this context, ***Carl Wilhelm Wutzer (1789–1863)***, a German surgeon practicing in Bonn, is mentioned. He had nothing to do with the operation itself, but at that time he was the head of the department where the procedure was performed.

### Intramedullary fixation of non-unions—1846–1861

The pioneer of intramedullary fixation was likely ***Johann Friedrich Dieffenbach (1792–1847)***, who practiced in Berlin. In 1846, he used intramedullary ivory pegs to treat non-unions of the femur, tibia, and humerus [37]. The method involved opening the medullary cavity with a drill and inserting an ivory peg. The peg served not so much for mechanical fixation as for biological stimulation, i.e., hyperaemia, which arose from infection of the surgical wound. The peg was removed one to three weeks after surgery, and the inflammatory hyperaemia led to healing of the non-union in some cases. In 1861, the German surgeon ***Theodor Billroth (1829–1894)*** treated a tibial non-union using a similar method [38]. Billroth removed the ivory pegs two weeks after the operation, examined them microscopically, and noted their partial resorption.

### Fractures of the femoral neck—1858

**Fig. 4:**
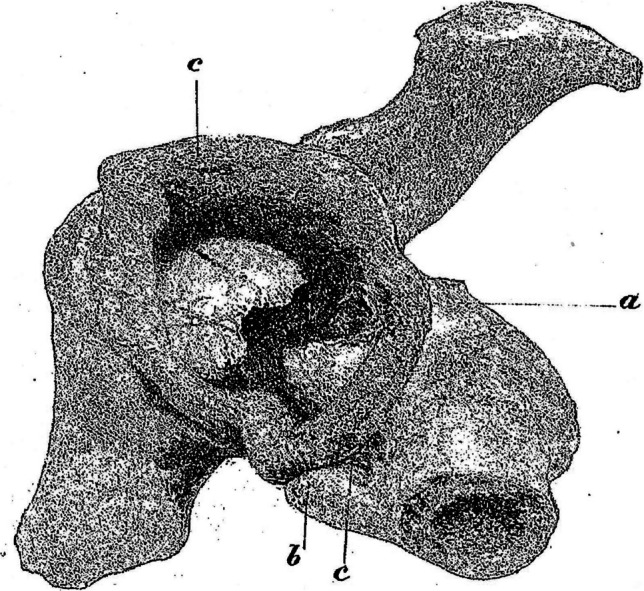
An autopsy specimen of a joint operated on by Langenbeck [39]. View of the open lower part of the joint capsule; the femoral head, the fracture line, and the femoral neck stump are visible

The most frequently discussed operatively treated fracture in the pre-aseptic era, particularly in the literature on the history of proximal femoral fractures [1, 3, 4, 6], was the femoral neck fracture treated by the renowned German surgeon ***Bernhard Rudolf Konrad von Langenbeck (1810–1887).*** For many years, information about this operation was cited from secondary sources and embellished with considerable imagination. Fortunately, the original description, published by Hugo Senftleben in 1858 [39], has been discovered. According to this description, Langenbeck treated a femoral neck non-union in a 50-year-old woman in January of that same year (1858). From an incision above the greater trochanter, he inserted an iron drill in the direction of the femoral neck to a depth of about 7.5 cm. However, an infection subsequently developed, requiring resection of the femoral head. Despite this, the patient died. The autopsy revealed the condition of the operated on hip joint (Fig. 4). This first surgery for a femoral neck fracture inspired other prominent European surgeons following the introduction of antisepsis. Langenbeck is also credited with the first use of external fixation. The case involved a humeral fracture treated with a simple external fixator in the late 1850s. However, further details are unknown [40].

### The end of pre-aseptic era—1870

Chief Medical Officer and Admiral of the French Imperial Navy ***Laurent Jean Baptiste Bérenger-Féraud (1832–1900)*** published an extensive monograph in 1870 titled *“Traité de l’immobilisation directe des fragments osseux dans les fractures”* [41], in which he summarized the cases and methods of internal fixation of fractures known at the time. He thus symbolically brought the pioneering era of surgical fracture treatment to a close. Despite a number of failures, individual surgeries served as inspiration for the next generation of surgeons. However, it took another 40 years before the Belgian surgeon ***Albin Lambott (1866–1955)*** laid the foundations of modern internal fixation of fractures [42, 43].

## Grant support

Supported by The Ministry of Defense, IP DZRVO MO1012.

## Dedication

This work is dedicated to the memory of our friend Prof. Ch. Colton (September 9, 1937 – December 24, 2025). Christopher Lewis Colton was an English orthopaedic surgeon and professor emeritus of orthopaedic and trauma surgery at the University of Nottingham. He served as president of the British Orthopaedic Association and the AO Foundation. In addition to orthopaedics, he was also passionate about the history of the field.

## Data Availability

No datasets were generated or analysed during the current study.
